# The ability of remaining glomerular podocytes to adapt to the loss of their neighbours decreases with age

**DOI:** 10.1007/s00441-022-03611-2

**Published:** 2022-03-15

**Authors:** James van der Wolde, Kotaro Haruhara, Victor G. Puelles, David Nikolic-Paterson, John F. Bertram, Luise A. Cullen-McEwen

**Affiliations:** 1 Department of Anatomy and Developmental Biology, Monash Biomedicine Discovery Institute, Monash University, Melbourne, Australia; 2 Division of Nephrology and Hypertension, Jikei University School of Medicine, Tokyo, Japan; 3 Department of Medicine, University Medical Center Hamburg-Eppendorf, Hamburg, Germany; 4 Departments of Nephrology and Medicine, Monash Health and Monash University, Clayton, Vic Australia

**Keywords:** Ageing, Kidney, Glomerulus, Podocyte, mTOR

## Abstract

Progressive podocyte loss is a feature of healthy ageing. While previous studies have reported age-related changes in podocyte number, density and size and associations with proteinuria and glomerulosclerosis, few studies have examined how the response of remaining podocytes to podocyte depletion changes with age. Mild podocyte depletion was induced in *Pod*^*Cre*^*iDTR* mice aged 1, 6, 12 and 18 months via intraperitoneal administration of diphtheria toxin. Control mice received intraperitoneal vehicle. Podometrics, proteinuria and glomerular pathology were assessed, together with podocyte expression of p-rp-S6, a phosphorylation target that represents activity of the mammalian target of rapamycin (mTOR). Podocyte number per glomerulus did not change in control mice in the 18-month time period examined. However, control mice at 18 months had the largest podocytes and the lowest podocyte density. Podocyte depletion at 1, 6 and 12 months resulted in mild albuminuria but no glomerulosclerosis, whereas similar levels of podocyte depletion at 18 months resulted in both albuminuria and glomerulosclerosis. Following podocyte depletion at 6 and 12 months, the number of p-rp-S6 positive podocytes increased significantly, and this was associated with an adaptive increase in podocyte volume. However, at 18 months of age, remaining podocytes were unable to further elevate mTOR expression or undergo hypertrophic adaptation in response to mild podocyte depletion, resulting in marked glomerular pathology. These findings demonstrate the importance of mTORC1-mediated podocyte hypertrophy in both physiological (ageing) and adaptive settings, highlighting a functional limit to podocyte hypertrophy reached under physiological conditions.

**Supplementary information:**

The online version contains supplementary material available at 10.1007/s00441-022-03611-2.

## Introduction

Podocytes are terminally differentiated epithelial cells with limited regenerative capacity. Following podocyte loss, remaining podocytes can hypertrophy in order to maintain the integrity of the glomerular filtration barrier, but the extent of this hypertrophy is limited and once reached podocytes detach resulting in proteinuria, glomerular tuft collapse and global glomerulosclerosis. The main clinical feature of the ageing kidney is reduced glomerular filtration rate (GFR) and loss of functioning nephrons (Denic et al. 2017; Hoy et al. 2003). Podocyte depletion is a characteristic of remaining glomeruli in ageing kidneys, rendering them susceptible to pathological change and ultimately loss (Brown et al. 2014; Dalla Vestra et al. 2003; Fukuda et al. 2012a, b; Kikuchi et al. 2015; Kriz et al. 1998, 2005; Lemley et al. 2002; Meyer et al. 1999; Pagtalunan et al. 1997; Sadowski et al. 2015; Steffes et al. 2001; Wang et al. 2009; Wharram et al. 2005; White et al. 2002; Wiggins 2007).

The mammalian target of rapamycin (mTOR) is critical for the regulation of metabolic processes within podocytes (Fantus et al. 2016). As well as its metabolic regulatory function, mTOR signalling has been implicated in the pathogenesis of various glomerular diseases including diabetic nephropathy (Inoki et al. 2011) and crescentic nephritis (Kurayama et al. 2011). Furthermore, constitutive mTOR activation is capable of inducing glomerular lesions in mice, whilst its partial blockade may ameliorate focal segmental glomerulosclerosis (FSGS)-like conditions (Zschiedrich et al. 2017). Conversely, podocyte-specific embryonic mTOR knockout mice display renal pathology (Gödel et al. 2011), and in the setting of a mild renal insult, mTOR-mediated podocyte hypertrophy plays an important adaptive role in mitigating any lasting glomerular damage (Puelles et al. 2019a). The importance of mTOR signalling for podocyte survival has also been demonstrated in the setting of chronic kidney disease (CKD) (Canaud et al. 2013). Taken together, the role of mTOR in the kidney appears to be a delicate balancing act with deviations in activity in either direction being potentially detrimental to kidney and, in particular glomerular, health.

It is well established that advanced age increases sensitivity to acute kidney injury, while decreasing the capacity of kidney tissue to repair (Chen et al. 2007; Denic et al. 2016; Wang et al. 2014; Xue et al. 2006). Surprisingly, however, few studies have reported an in-depth examination of age-related glomerular changes, and fewer still have assessed the changing adaptive capacity of podocytes with age. Recently, Schneider et al. (2017) assessed the compound effects of age and podocyte depletion in an experimental model of FSGS on parietal epithelial cells and, to a lesser degree, podocytes (Schneider et al. 2017). However, to date, no study has investigated age-related changes in the adaptive potential of podocytes. Here, we describe age-related changes in podometrics and assess the adaptive capacity of remaining podocytes following mild podocyte depletion. We also investigate the role of podocyte mTOR signalling in ageing kidneys with podocyte depletion.

## Materials and methods

### Animal model

Animal experiments were conducted in accordance with the Monash Animal Research Platform guidelines (ethics approval number: MARP/2014/015) using male ROSA26^iDTR/iDTR^ (iDTR, imported from JAX Laboratories) or Pod^Cre^; ROSA26^iDTR/iDTR^ mice (Pod^Cre^ mice provided by Dr. Susan Quaggin, Feinberg Cardiovascular Research Centre). Confirmation of Cre expression was determined via PCR utilising genomic DNA obtained from tail biopsies. The Cre allele was tested for using primer pairs: 59-GCGGTCTGGCAGTAAAAACTATC-39 (Cre-F) and 59-GTGAAACAGCATTGCTGTCACTT-39 (Cre-R). An internal control (IL-2) was also assessed using primer pairs: 59-CTAGGCCACAGAATTGAAAGATCT-39 (IL-2F) and 59-GTAGGTGGAAATTCTAGCATCATCC-39 (IL-2R). Mice allocated to control (*n* = 13 at 1 month, *n* = 16 at 6 months, *n* = 15 at 12 months and *n* = 9 at 18 months) or DT administration (*n* = 12 at 1 month, *n* = 16 at both 6 and 12 months and *n* = 8 at 18 months) were matched for bodyweight and 3-h fasting blood glucose (Supplementary Table 1). Fasting glucose was tested using a tail venous blood sample on an Accu-Check® Mobile (Hoffman-La Roche, Basel, Switzerland) blood glucose monitor.

### DT administration

Pod^Cre^iDTR mice aged 1, 6, 12 and 18 months were injected intraperitoneally with a DT dose of 0.05 μg/kg body weight. Pod^Cre^iDTR mice injected with saline at these same ages served as controls. We have previously shown that this DT dose results in loss of approximately 15% of podocytes in mice aged 1 month (Puelles et al. 2016b). To examine if additional podocyte loss and hypertrophy occurred following the initial DT induced injury, control and DT-injected mice aged 1 month at time of DT administration were euthanised either 1 or 4 weeks after DT administration. No statistical differences were identified and data sets were combined. Similarly, mice aged 6, 12 and 18 months at time of DT injection were euthanised either 2 or 8 weeks later. No statistical difference was identified between mice collected at 2- and 8-week post-DT, and data sets were combined. Following perfusion fixation, kidneys were sliced at 800 μm using a razor blade slicing device. One mid-hilar slice was processed and embedded in paraffin for histopathology (see below), and another slice was processed for immunofluorescence labelling and podometrics (see below).

### Histopathology

One 2-μm mid-hilar histological section of each kidney was stained with periodic acid-Schiff (PAS) and imaged using an Aperio ScanScope FL (Leica Biosystems, Wetzlar, Germany) at 40 × magnification. Every glomerulus (median number of 95 glomeruli per section) in these sections was viewed and scored for sclerosis to give a glomerulosclerotic index (GSI). A score of 0 was assigned to normal glomeruli, a score of 1 if glomerulosclerosis, mesangial expansion or periglomerular infiltration was present in 1–25% of the glomerulus, a score of 2 if these factors were present in 26–50% of the glomerulus, a score of 3 if these factors were present in 51–75% of the glomerulus and a score of 4 if these factors were present in 76–100% of the glomerulus. GSI was calculated using the formula:

$$\mathrm{GSI}=\frac{\left[\left(1\times N1\right)+\left(2\times N2\right)+\left(3\times N3\right)+\left(4\times N4\right)\right]}{\mathrm N0+\mathrm N1+\mathrm N2+\mathrm N3+\mathrm N4}$$

where *N* is the number of glomeruli with each given score for a given section.

Diffuse glomerular lesion (mesangial expansion; ME) in each glomerulus in the PAS section was evaluated and scored as follows: 0 normal or mild mesangial expansion, 1 mesangial expansion ≤ capillary lumen, 2 mesangial expansion = capillary lumen, 3 mesangial expansion ≥ capillary lumen. ME was calculated using the formula:

$$\mathrm{ME}=\frac{\left[\left(1\times N1\right)+\left(2\times N2\right)+\left(3\times N3\right)\right]}{\mathrm N0+\mathrm N1+\mathrm N2+\mathrm N3}$$

where *N* is the number of glomeruli with each given score for a given section.

Periglomerular infiltration (PGI) in each glomerulus was evaluated as 0 (absent) or 1 (present), and the periglomerular infiltration score was calculated by dividing the number of glomeruli affected by the total number of glomeruli in the section.

### Immunofluorescence labelling for podometrics

One 800-μm-thick slice from each kidney was immunostained for podometric analysis as previously described (Puelles et al. 2016b). In short, slices were subject to a 1-h antigen retrieval protocol at 98 °C for 1 h using Dako Target Retrieval Solution (S1699; Dako, Glostrup, Denmark) in a Dako PT Link Instrument (PT10126; Dako). Slices were then incubated for 6 days at 37 °C in a primary antibody solution containing polyclonal rabbit anti-mouse p57 (1:200; SC8298; Santa Cruz Biotechnology, Santa Cruz, CA) and polyclonal goat anti-mouse SNP (1:400; SC21537; Santa Cruz Biotechnology). All antibodies were diluted in Dako Antibody Diluent (S0809; Dako). Slices were then washed in 4 ml of Dako Wash Buffer (K8007; Dako) at 37 °C for 24 h. Slices were then immersed in a secondary antibody solution containing polyclonal donkey anti-rabbit Alexa Fluor® 555 (1:200; A31572; Life technologies, Carlsbad, CA) and polyclonal chicken anti-goat Alexa Fluor® 488 (1:200; A21467; Life technologies) and incubated for 6 days at 37 °C, protected from light. Finally, slices were washed in 4 ml of Dako Wash Buffer (K8007; Dako) at 37 °C for 24 h. A full list of antibodies used in this study may be found in Supplementary Table 2.

### Optical clearing

Immunostained slices were placed flat in a glass petri dish and covered with a 2% solution of analytical grade low-melting point agarose (V2111; Promega, Fitchburg, WI) in water. Once set, slices were subjected to a serial dehydration protocol; 2 h in 50% EtOH in water, 2 h in 70% EtOH in water, 2 h in 100% EtOH and 24 h in 100% EtOH. All EtOH was then removed, and cyanoacrylate super glue was used to adhere the edges of the agarose disc to the petri dish prior to clearing with ethyl cinnamate (1 12,372; Sigma-Aldrich). The agarose disc and embedded tissue became clear within 3 h.

### Confocal imaging

**Fig. 1:**
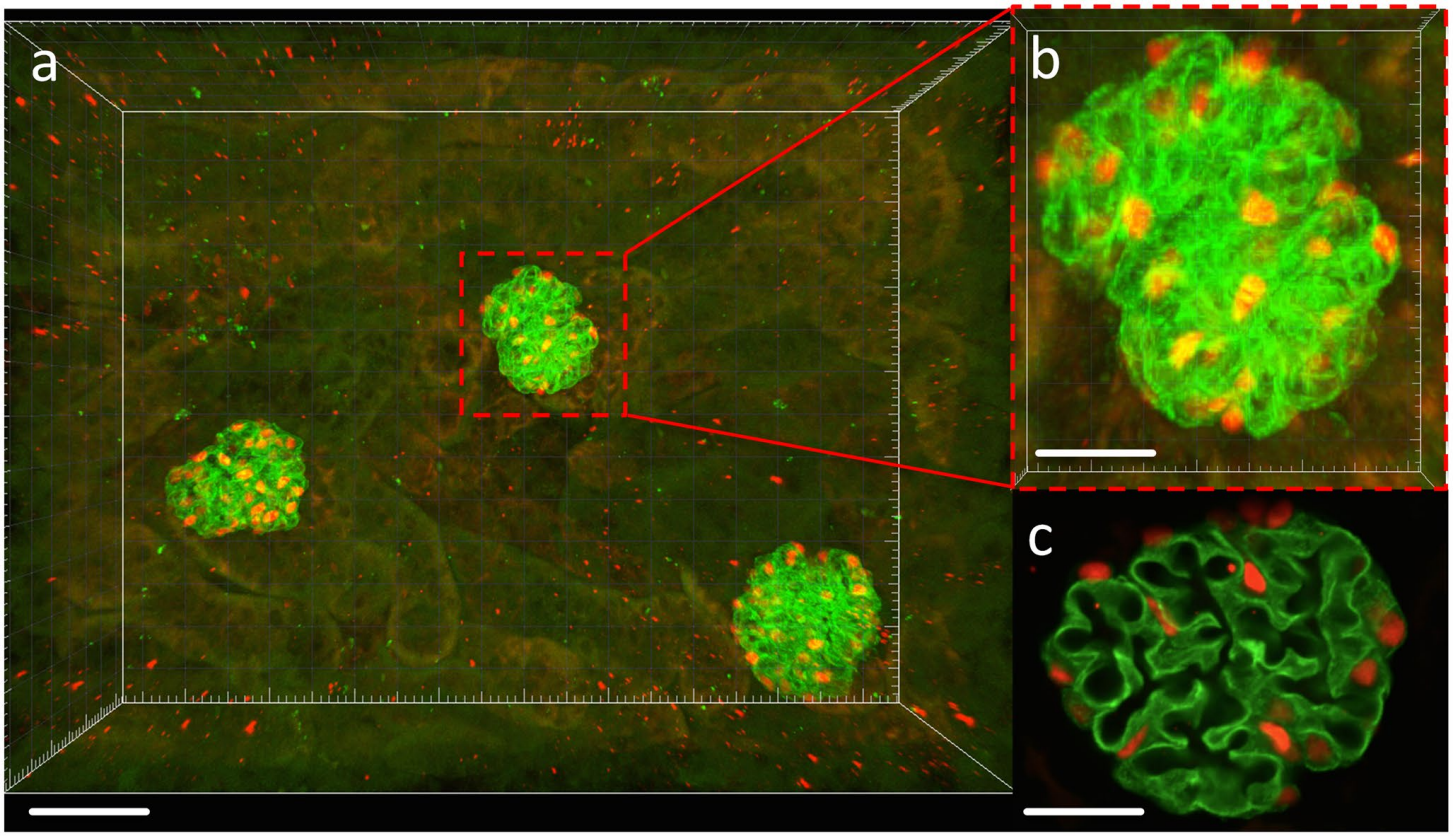
Glomerular and podocyte imaging. **a** Three-dimensional image field containing three whole glomeruli showing immunofluorescence labelling and identification of podocytes. **b** Cropped whole glomerulus sampled for podometric analysis. **c** Confocal section through a whole glomerulus. Synaptopodin (green), p57 (red). Scale bars: **a** 60 μm; **b**, **c** 20 μm

Twenty whole glomeruli in each slice were imaged using a Leica SP8 Multiphoton Microscope (Leica Microsystems, Wetzlar, Germany) fitted with a 20 × BABB immersion objective lens (numerical aperture, 0.95; working distance, 1950 μm). Serial optical sections were imaged utilising a z-step size of 1 μm with image series captured at a resolution of 1024 × 1024 pixel frames (Fig. 1). Imaging of 4-μm paraffin-embedded sections was completed on a Leica SP5 Microscope (Leica Microsystems) fitted with a 40 × oil immersion objective lens (numerical aperture 1.25). Representative images were captured at variable zoom at a resolution of 1024 × 1024 pixel frames.

### Quantification of podocyte and glomerular indices

Podocyte quantification was completed with the assistance of Fiji imaging software (Max Planck Institute of Molecular Cell Biology and Genetics, Dresden, Germany) as previously described (Puelles et al. 2016b). In short, podocyte nuclei were identified based on positive p57 expression. Image stacks were scrolled through and podocyte nuclei were counted in 20 whole glomeruli per animal utilising the built-in cell counter feature (https://imagej.nih.gov/ij/plugins/cell-counter.html). Podocyte three dimensional indices were calculated as previously described (Puelles et al. 2016b). Briefly, podocyte volumes were estimated using the 3D rendering software Imaris, version 9 (Bitplane, Belfast, Ireland) based on SNP + and p57 + voxels. Estimation of glomerular and podocyte volume was performed using 3-D rendering and analysis software (Imaris, version 8; Bitplane AG) as previously reported (Puelles et al. 2016b).

### p-rp-S6 immunofluorescence

Four-micrometer paraffin-embedded sections were stained in accordance with previously published methods (Puelles et al. 2019b). In short, sections were subject to a 30-min antigen retrieval protocol at 98 °C using Dako Target Retrieval Solution (S1699; Dako) in a Dako PT Link Instrument (PT10126; Dako). Slices were left to cool then incubated overnight at 4 °C in a primary antibody solution containing polyclonal rabbit anti-mouse phosphor-r6 Ribosomal Protein (1:50; 5364S; Cell Signal Technology, Danvers, MA) and polyclonal goat anti-mouse SNP (1:400; SC21537; Santa Cruz Biotechnology. Sections were washed 5 × for 5 min with Dako Wash Buffer (K8007; Dako). Sections were then incubated in a secondary antibody solution containing polyclonal donkey anti-rabbit Alexa Fluor® 555 (1:200; A31572; Life technologies) and polyclonal chicken anti-goat Alexa Fluor® 488 (1:200; A21467; Life technologies) for 1 h at room temperature, protected from light. Sections were washed 5 × for 5 min with Dako Wash Buffer (K8007, Dako) and incubated for 20 min with DAPI (1:10,000; D1306; Life Technologies, Carlsbad, CA). Sections were washed 5 × for 5 min with Dako Wash Buffer (K8007, Dako) before being coverslipped using Prolong Gold (P36934; Invitrogen, Carlsbad, CA).

### Albumin to creatinine ratio

Urine was collected on day 0 prior to injection of saline (control) or DT and then on days 5, 8, 15, 23, 30, 42, and 56 after DT administration and stored at − 80 °C. Urinary albumin concentration was calculated using the Albuwell M Albumin ELISA (1011; Exocell, Philadelphia, PA), and urinary creatinine concentration was calculated using The Creatinine Companion (1012; Exocell). An ACR ratio was then generated.

### Statistical analysis

Statistical analyses were performed using GraphPad Prism 8 (GraphPad software, La Jolla, CA). Each data set was subject to a ROUT outlier test (*Q* = 1% false discovery rate; outliers were removed) and D’Agostino-Pearson normality test. Due to the variable nature of ACR and GSI data, ROUT testing was not completed on these data sets. ACR data were log transformed to achieve normality prior to analysis. The effect of podocyte depletion and age was analysed by two-way ANOVA. Unless otherwise stated, data are presented as means ± SEMs with *p* values adjusted for multiple comparisons using Sidak correction. Associations between variables were determined using Spearman’s rank correlation coefficient. Statistical significance was defined as *p* < 0.05.

## Results

### Intra-individual variability within the 20 sampled glomeruli per mouse

Twenty whole individual glomeruli were analysed per mouse for three podocyte indices (podocyte number, volume and density). To determine if DT treatment and/or age influenced the within-mouse variability (coefficient of variation, CV) for these parameters, CVs were calculated for each mouse and compared between groups (control, DT) and across time (1, 6, 12 and 18 months). DT treatment did not affect variability within mice for either podocyte number (*p* = 0.21), volume (*p* = 0.80) or density (*p* = 0.48). However, the variability in podocyte number within mice increased significantly between 1 and 18 months in both control (*p* < 0.01) and DT-treated mice (*p* < 0.01), and there was a similar trend for podocyte volume (*p*_time_ = 0.06) and podocyte density (*p*_time_ = 0.07).

### Glomerular volume and podometrics changed dramatically with age in control mice

**Fig. 2:**
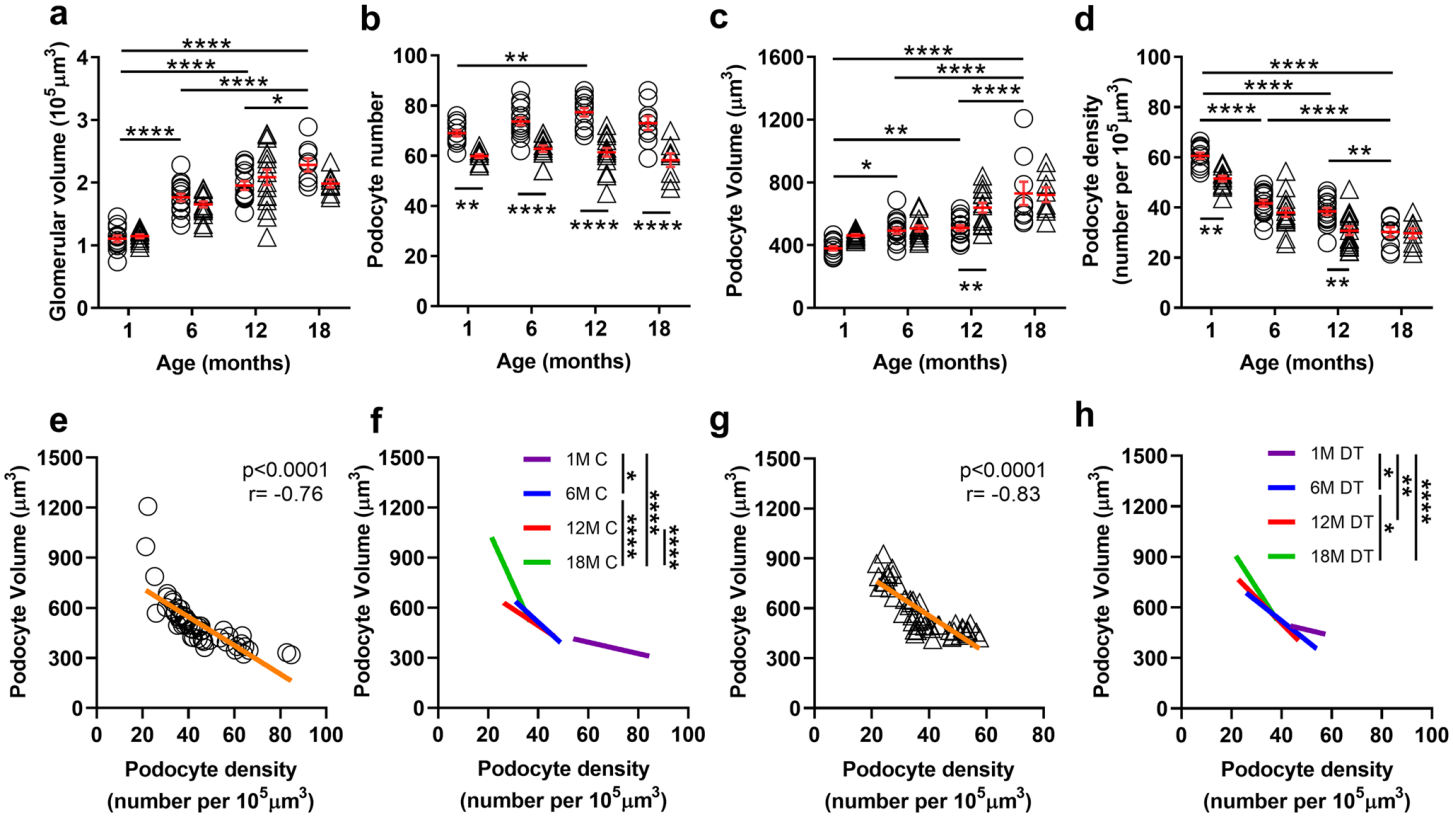
Glomerular volume and podometric indices in control and DT-injected mice aged 1, 6, 12 and 18 months. Glomerular volume **a**. Podocyte number per glomerulus **b**. Podocyte volume **c**. Podocyte density (number of podocytes per unit volume of glomerulus) **d**. Each symbol represents a mouse; circles, controls; triangles, DT injected. Mean values and standard errors indicated in red. Data analysed by two-way ANOVA (time and treatment) where ****p < 0.0001, **p < 0.01, *p < 0.05 following adjustment for multiple comparisons. Significant differences between control and DT groups are indicated below data points; significance differences across age are indicated above data points. Podocyte density and average podocyte volume regression slope analysis in control mice across the age span **e** and for each timepoint with different age groups shown in different colours **f**. Podocyte density and average podocyte volume regression slope analysis in DT injected mice across the age span **g** and for each timepoint with different age groups shown in different colours **h**. Data in **f** and **h** analysed by two-way ANOVA where ****p < 0.0001, ***p < 0.001, **p < 0.01, *p < 0.05 following adjustment for multiple comparisons

Glomerular and podometric data are presented in Fig. 2 and Supplementary Table 3. As expected, glomerular volume increased significantly with age, more than doubling between 1 and 18 months of age (*p* < 0.0001, Fig. 2a). Most of this glomerular hypertrophy in control mice (60%) occurred between 1 and 6 months of age (*p* < 0.0001). No change in glomerular volume occurred between 6 and 12 months, but a further 17% increase in glomerular volume occurred between 12 and 18 months (*p* < 0.02).

Podocyte number per glomerulus was similar at 1, 6 and 18 months (1 month, 69 ± 1; 6 months, 74 ± 2; 18 months, 73 ± 3 podocytes per glomerulus) in control mice (Fig. 2b). Interestingly, podocyte number at 12 months (77 ± 2) was significantly higher than at 1 month of age (*p* < 0.01; Fig. 2b). The largest podocytes were found in the oldest mice (18 months; Fig. 2c), with podocyte volume almost doubling between 1 and 18 months (76% increase, *p* < 0.0001). Podocyte volume increased by 29% between 1 and 6 months (*p* < 0.05), did not change between 6 and 12 months and then increased by a further 32% between 12 and 18 months (*p* < 0.0001; Fig. 2c). Podocyte density more than halved between 1 and 18 months of age in control mice (*p* < 0.0001), with a 31% decrease occurring between 1 and 6 months (*p* < 0.0001), no change between 6 and 12 months and a 21% decrease between 12 and 18 months (*p* < 0.01; Fig. 2d).

### The impact of podocyte loss on podometrics varied with age

Glomerular and podometric data are presented in Fig. 2 and Supplementary Table 3. DT administration did not alter glomerular volume at any timepoint (*p*_treatment_ = 0.30; Fig. 2a). As expected, DT administration resulted in significant podocyte loss at each timepoint (*p*_treatment_ < 0.0001), being 13% at 1 month, 15% at 6 months and 20% at both 12 and 18 months (Fig. 2b). Although podocyte loss appeared higher at 12 and 18 months, this difference did not reach statistical significance (*p*_treatment*time_ = 0.13).

Despite significant DT-induced podocyte loss at 1 and 6 and months of age, the volume of remaining podocytes was similar to that in age-matched control mice. However, this was not the case at 12 months of age, when remaining podocytes in DT-treated mice were 30% larger than podocytes in age-matched controls (*p* < 0.001; Fig. 2c). However, when DT was administered at 18 months, remaining podocytes did not undergo adaptive hypertrophy.

DT administration and the resulting podocyte loss resulted in a 15% decrease in podocyte density at 1 month (*p* < 0.01) and a 20% decrease at 12 months (*p* < 0.01). In contrast, podocyte density did not change following podocyte loss at 6 (*p* = 0.26) or 18 months of age (*p* = 0.99) (Fig. 2d).

### The relationship between podocyte density and volume was similar in control mice and mice with podocyte depletion, but changed with age

Given the striking age-associated changes in podocyte volume and density, we assessed the relationship between podocyte volume and density across the age span. As expected, podocyte volume in control mice was inversely correlated with podocyte density at 1 (*p* < 0.01; *r* = − 0.70), 6 (*p* < 0.0001; *r* = − 0.89), 12 (*p* < 0.001; *r* = − 0.79) and 18 months (*p* < 0.002; *r* = − 0.89) (Supplementary Fig. 1a–d). Similar findings were observed following administration of DT and the resulting podocyte depletion (Supplementary Fig. 1e–h; 1 month, *p* = 0.08; 6 months, *p* < 0.01; *r* = − 0.68; 12 months, *p* < 0.001; *r* = − 0.80 and 18 months, *p* < 0.0001; *r* = − 0.96).

Across the 18-month age span, podocyte volume in control mice increased by 8.6 µm^3^ for every unit decrease in podocyte density (*p* < 0.0001; *r* = − 0.76; Fig. 2e). Interestingly, the rate of increase in podocyte volume per unit decrease in podocyte density varied almost tenfold between 1 and 18 months of age in control mice, being just 3.4 µm^3^ at 1 month, 13.7 µm^3^ at 6 months, 9.9 µm^3^ at 12 months and 33.2 µm^3^ at 18 months (Supplementary Fig. 1a–d). Regression lines for the four timepoints are shown in Fig. 2f. Comparison of the regression slopes showed that the rate of increase in podocyte volume (as podocyte density decreases) rose significantly between 1 and 6 months (*p* < 0.05) and again between 12 and 18 months (*p* < 0.0001) (Fig. 2f) in control mice.

The rate of increase in podocyte volume per unit decrease in podocyte density in DT-treated mice was similar to that in control mice (*p*_treatment_ = 0.42; 11.3 µm^3^ increase in podocyte volume per unit decrease in podocyte density; *p* < 0.0001; *r* = − 0.83; Fig. 2g), being 3.5 µm^3^ at 1 month, 11.8 µm^3^ at 6 months, 15.7 µm^3^ at 12 months and 23.2 µm^3^ at 18 months (Supplementary Fig. 1e–h). Comparison of the regression slopes showed the rate of increase in podocyte volume (as podocyte density decreases) rose significantly between 1 and 6 months (*p* < 0.05) and again between 6 and 18 months (Fig. 2h).

### Albumin excretion increased with age in control mice

**Fig. 3:**
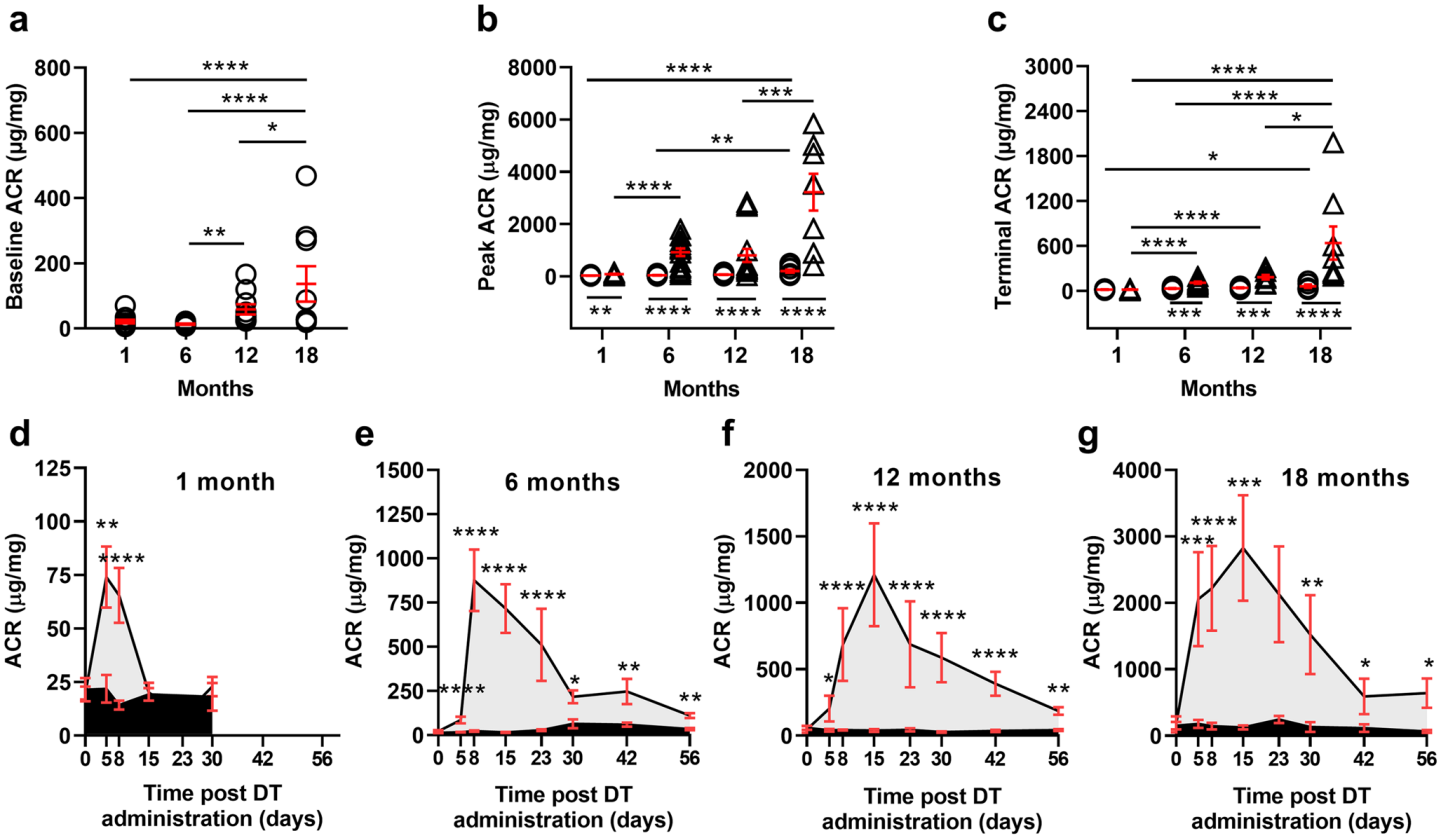
Albumin excretion in control and DT-injected mice aged 1, 6, 12 and 18 months. **a** Baseline (day 0), **b** peak and **c** terminal ACR levels in mice aged 1, 6, 12 and 18 months at time of vehicle or DT injection. Albumin excretion across the 8-week experimental period for mice aged **d** 1, **e** 6, **f** 12 and **g** 18 months at time of DT injection. *n* ≥ 8 mice in each group. Data analysed by two-way ANOVA where ****p < 0.0001, ***p < 0.001, **p < 0.01, *p < 0.05 following adjustment for multiple comparisons. Statistically significant differences between control and DT groups in **a, b** and **c** indicated below data points; significant differences across time in **a, b** and **c** indicated above data points. Circles, controls. Triangles, DT-injected. Each symbol represents a mouse. Mean values and standard errors indicated in red

Baseline (day 0 for all animals in each of the four age groups) urinary albumin excretion was similar at 1 and 6 months of age, but increased significantly between 6 and 12 months of age and again between 12 and 18 months of age (Fig. 3a and Supplementary Table 4).

### The impact of podocyte depletion on urinary albumin excretion increased with age

The impact of DT administration on urinary albumin excretion increased markedly with age, reaching its peak in mice in the oldest age group (18 months). In brief, in 1-month mice, ACR peaked at 74 μg/mg 5 days after DT administration and returned to control levels by day 15 (Fig. 3b and d). In comparison, albuminuria peaked at 8 days post-DT administration in 6-month mice at approximately 900 μg/mg (Fig. 3b and e) and 15 days after DT administration in 12- and 18-month mice at 1200 and 2800 μg/mg, respectively (Fig. 3b, f, and g). Peak and terminal ACR for each mouse for each age are shown in Fig. 3b and c, respectively. Two-way ANOVA showed a significant interaction between age and DT treatment on both peak (*p* < 0.001; Fig. 3b) and terminal albumin excretion (*p* < 0.001; Fig. 3c) clearly indicating that the effect of DT administration on albuminuria was markedly affected by age.

### Glomerular pathology in control mice

**Fig. 4:**
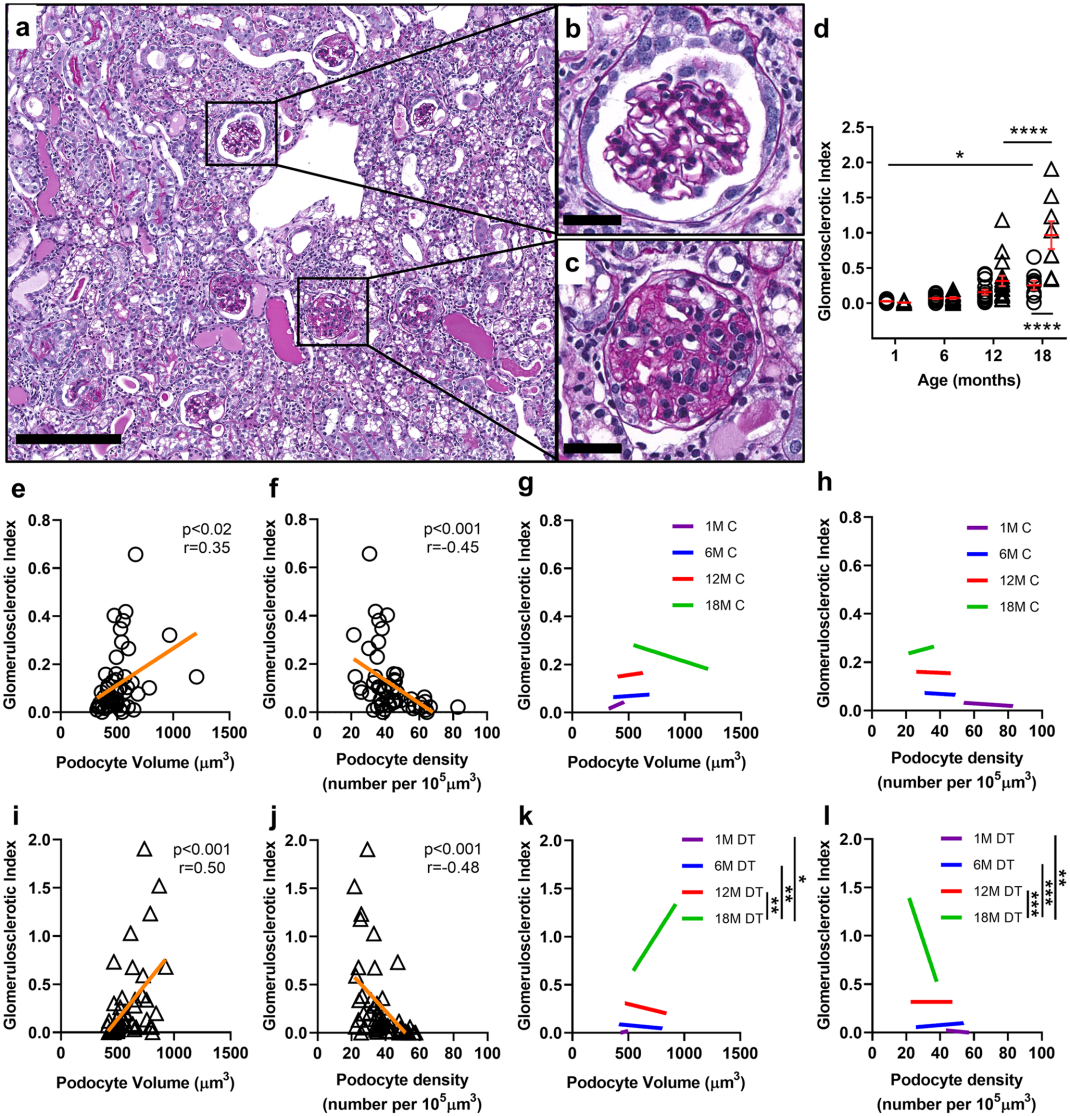
Glomerulosclerosis in control and DT-injected mice aged 1, 6, 12 and 18 months. Representative PAS-stained section used for analysis of glomerulosclerotic index **a** (DT-injected, 18-month-old mouse). Glomerulus with open capillaries with a GSI score of 0 **b** compared to glomerulus with sclerotic lesion and a GSI score of 4 **c**; Scale bar = 200 µm **a** and 25 µm **b** and **c**. GSI in control and DT-injected mice aged 1, 6, 12 and 18 months **d**. Circles, controls. Triangles, DT-injected. Each symbol represents a mouse. Mean values and standard errors indicated in red. Data analysed by two-way ANOVA where ****p < 0.0001, *p < 0.05 following adjustment for multiple comparisons. Statistically significant differences between control and DT groups are indicated below data points; significant differences between age groups are indicated above data points. Correlation between GSI and podocyte volume **e** and podocyte density **f** in control mice across the age span. Regression slope analysis of GSI and podocyte volume **g** and podocyte density **h** in control mice across the age span. Correlation between GSI and podocyte volume **i** and podocyte density **j** in DT injected mice across the age span. Regression slope analysis of GSI and podocyte volume **k** and podocyte density **l** in DT injected across the age span. Dark purple line, 1 month; blue line, 6 months; red line, 12 months; green line, 18 months. Data in **g**, **h**, **k**, and **l** analysed by two-way ANOVA where ***p < 0.001, **p < 0.01, *p < 0.05 following adjustment for multiple comparisons

PAS staining was used to analyse pathology at each time point (Fig. 4a, b, and c). GSI increased slightly between 1 and 18 months of age in control mice (*p* < 0.05; Fig. 4d).

No significant relationships were observed between glomerulosclerosis and podocyte volume (Supplementary Fig. 2a–d) or podocyte density (Supplementary Fig. 2e–h) in control mice at any age. However, when control values for all four timepoints were combined, glomerulosclerosis was found to be directly correlated with podocyte volume (*r* = 0.35; *p* < 0.01; Fig. 4e) and inversely correlated with podocyte density (*r* = − 0.45; *p* < 0.001; Fig. 4f). Regression slopes for each timepoint are shown in Fig. 4g and h). Interestingly, the rate of increase in glomerulosclerosis with increasing podocyte volume and decreasing podocyte density did not change with age in control mice.

In control mice, mesangial expansion increased from 6 to 12 months of age (*p* < 0.0001) but remained stable between 12 and 18 months (Supplementary Fig. 3a). The periglomerular infiltration index was similar in control mice at all ages (Supplementary Fig. 3b).

### Podocyte depletion at 18 months resulted in a fourfold increase in glomerulosclerosis

There was no increase in GSI following mild podocyte depletion at 1, 6 or 12 months of age (Fig. 4d). However, 18-month-old mice showed a significant increase in GSI in response to mild podocyte depletion (*p* < 0.0001) with GSI fourfold higher than in control mice, and also 4 times higher than both control and DT-treated mice at 12 months (*p* < 0.0001 in both cases; Fig. 4d).

The relationship between GSI and podocyte volume and podocyte density in DT-treated mice is shown in Supplementary Fig. 2e–h and m–p, respectively. Across the 18-month age span podocyte volume in DT-treated mice increased as GSI increased (*p* < 0.0001; Fig. 4i), while podocyte density decreased as GSI increased (*p* < 0.001; Fig. 4j). In both cases, the rate of increase in GSI was markedly higher at 18 months than at younger ages (Fig. 4k and l).

Podocyte depletion did not alter the degree of mesangial expansion at any age (Supplementary Fig. 3a). DT-induced podocyte depletion increased periglomerular infiltration at 12 months, but this did not reach statistical significance (*p* = 0.09). However, 18-month mice with podocyte depletion showed greater periglomerular infiltration than age-matched controls (*p* < 0.001) as well as DT-treated mice at 12 months of age (*p* < 0.01; Supplementary Fig. 3b).

### Podocyte mTORC1 expression increased with age in control mice

**Fig. 5:**
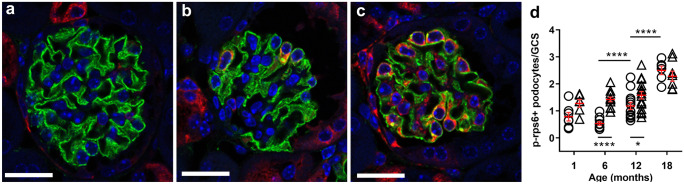
Podocyte p-rp S6 expression in control and DT-injected mice aged 1, 6, 12 and 18 months. Representative images of glomeruli in DT-injected 6-month mice with no **a**, mild **b** and high **c** numbers of podocytes expressing p-rp-S6. Number of p-rp-S6 + podocytes per glomerulus in control and DT-injected mice aged 1, 6, 12 and 18 months **d**. ****p < 0.0001, *p < 0.05 following adjustment for multiple comparisons. Statistically significant differences between control and DT groups are indicated below data points; significant differences across time are indicated above data points. Circles, controls. Triangles, DT-injected. GCS, glomerular cross-section

Podocyte mTORC1 expression was evaluated via immunofluorescence staining for phosphorylated ribosomal protein S-6, a downstream component of the mTORC1 signalling pathway (Fig. 5a–c). In control mice, the number of mTOR + podocytes per glomerular cross-section did not change between 1 and 6 months of age, but doubled between 6 and 12 months (*p* < 0.0001) and doubled again between 12 and 18 months (*p* < 0.0001; Fig. 5d).

*Reduced mTORC1 activation despite of significant podocyte depletion in older mice* Administration of DT significantly increased the number of mTOR + podocytes per glomerular cross-section at 6 and 12 months. This increase was most marked at 6 months when a 168% increase above control levels was observed (*p* < 0.0001). At 12 months, DT administration increased the number of mTOR + podocytes by 33% (*p* < 0.05). A 63% increase in the number of mTOR + podocytes per glomerular cross-section following DT administration was observed in 1-month mice, but this increase was not statistically significant (*p* = 0.08). However, unlike the other three age groups, DT administration had no effect on the number of mTOR + podocytes per glomerular cross-section in mice aged 18 months (*p* = 0.35; Fig. 5d).

## Discussion

The major findings from this study on the individual and combined effects of healthy ageing and mild podocyte depletion on podometrics, albuminuria and glomerulosclerosis are as follows: (i) while podocyte number per glomerulus did not change in control mice in the 18-month time period examined, control mice at 18 months had the largest podocytes, the lowest podocyte density and evidence of podocyte functional decline; (ii) while DT administration resulted in similar levels of podocyte depletion at the four timepoints, mice at 1, 6 and 12 months developed mild albuminuria but no glomerulosclerosis, whereas 18-month mice had high levels of albuminuria and glomerulosclerosis; and (iii) unlike younger mice, 18-month mice with mild podocyte depletion did not show an increase in the number of mTOR + podocytes per glomerulus which may have limited their ability to adapt to podocyte loss. These findings are discussed below.

Our finding of a significant decrease in podocyte density with age in control mice confirms findings from previous studies (Kaverina et al. 2020; Pippin et al. 2014; Roeder et al. 2015; Schneider et al. 2017; Sweetwyne et al. 2017). A decrease in podocyte density can result from podocyte loss and/or glomerular hypertrophy. In the present study, no change in podocyte number per glomerulus was observed in control mice between 1 and 18 months, indicating that the observed age-related decrease in podocyte density was entirely due to the more than twofold increase in glomerular volume in this period. Interestingly, Kaverina et al. (2020) also reported no loss of podocytes in healthy glomeruli of middle-aged (14 months) and aged (20–24 months) mice (Kaverina et al. 2020). Our findings are also in agreement with those of Wiggins et al. (2005) who found no podocyte loss in Fischer 344 rats between 2 and 24 months of age (Wiggins et al. 2005).

In humans, podocyte density decreases with age due to a combination of podocyte loss and glomerular enlargement. Puelles et al. (2016a, b) reported that mean glomerular volume increases sevenfold between childhood and adulthood (Puelles et al. 2016a). Hodgin et al. (2015) reported that podocyte density decreased from > 300 per 10^6^ µm^3^ of glomerular volume in subjects aged less than 20 years to < 100 per 10^6^ µm^3^ in subjects aged 70–80 years, a rate of decrease of approximately 0.9% per year (Hodgin et al. 2015).

Age-related podocyte loss has been reported in humans and can thus contribute to decreased podocyte density. Normal adult human glomeruli contain approximately 500–600 podocytes each (Kikuchi et al. 2017; Puelles et al. 2016a). Puelles et al. (2016a, b) reported that glomeruli in older subjects (39–74 years of age) contained 17% fewer podocytes than glomeruli in younger subjects (18–29 years of age) (Puelles et al. 2016a). Hodgin et al. (2015) reported that podocyte number per glomerulus decreased from about 550 in children to approximately 400 podocytes per glomerulus in subjects aged in their 80 s (Hodgin et al. 2015). A range of mechanisms have been implicated in age-associated podocyte depletion including apoptosis (Lee et al. 2004; Tower 2015), pyroptosis (Shankland et al. 2021), podocyte detachment (Hodgin et al. 2015; Yu et al. 2005) and mitotic catastrophe (Lasagni et al. 2013).

The decrease in podocyte density in control mice in the present study was associated with an increase in podocyte volume, with 18-month podocytes on average being almost twice the size of podocytes at 1 month. This podocyte hypertrophy successfully maintained the integrity of the glomerular filtration barrier at 6 months with no albuminuria or glomerulosclerosis observed. However, mild albuminuria was observed from 12 months, and glomerulosclerosis was evident at 18 months. These findings suggest that by 18 months of age, podocytes in control mice were struggling to maintain the integrity of the glomerular filtration barrier. An association between reduced podocyte density and increased podocyte volume has previously been reported in humans and mice. Wiggins et al. described the stages of podocyte hypertrophy that include non-stressed hypertrophy with normal function, adaptive hypertrophy in which podocyte function remains normal despite signs of stress and overt proteinuria is not observed, decompensated hypertrophy when podocytes are stressed and beginning to lose function and proteinuria is evident and a final stage when podocytes fail to hypertrophy sufficiently to maintain barrier integrity leading to relative and absolute podocyte depletion and consequent glomerulosclerosis (Wiggins et al. 2005). Based on these stages, the 12-month control podocytes in the present study appear to be in the decompensated stage because their density has not changed from 6 months but proteinuria is evident. In contrast, relative podocyte depletion is present at 18 months in control mice, and both proteinuria and glomerulosclerosis are also present. These 18-month kidneys are thus extremely vulnerable to any additional podocyte loss that might occur.

Based on these findings in control mice at 18 months, it is not surprising that the impact of DT-induced podocyte depletion on glomerular barrier function and the development of glomerulosclerosis were most marked at this timepoint. There is ample evidence that podocyte depletion, either absolute or relative, is an early event in the development of glomerulosclerosis (Brown et al. 2014; Dalla Vestra et al. 2003; Fukuda et al. 2012a, b; Kikuchi et al. 2015; Kriz et al. 1998; Kriz et al. 2005; Lemley et al. 2002; Meyer et al. 1999; Pagtalunan et al. 1997; Sadowski et al. 2015; Steffes et al. 2001; Wang et al. 2009; Wharram et al. 2005; White et al. 2002; Wiggins 2007). There is also evidence that the development of age-associated glomerulosclerosis is associated with age-associated decreases in podocyte number and density (Hodgin et al. 2015; Puelles et al. 2016a; Wiggins et al. 2005).

Of relevance here is the study by Schneider et al. 2017 who studied the additive effects of disease-induced podocyte loss (via the use of a cytopathic anti-podocyte antibody) and age-associated podocyte depletion in a mouse model of FSGS (Schneider et al. 2017). Studies in mice aged 3 and 27 months showed that compared to younger FSGS mice, aged FSGS mice had lower podocyte density, higher parietal cell activation, migration and epithelial to mesenchymal transition and increased collagen IV staining in Bowman’s capsule. Interestingly, a more severe depletion of podocytes was observed in young FSGS mice than in aged FSGS mice. Schneider et al. (2017) interpreted this finding as reflecting the fact that the younger mice had a much higher podocyte density from the outset (Kriz et al. 1998). In contrast, to the findings of Schneider et al. (2017), we found that DT administration resulted in a similar level of podocyte loss at the four timepoints, although we did not study mice at 27 months. These differences in findings between studies may also reflect the use of different toxins to deplete podocytes and/or the use of podocyte densities versus absolute podocyte number to measure podocyte loss.

While the present findings clearly illustrate the heightened vulnerability of the ageing kidney to podocyte depletion and the subsequent development of proteinuria and glomerulosclerosis, the mechanisms whereby age increases podocyte susceptibility to disease remain unclear. In their recent timely review on podocyte ageing, Shankland et al. (2021) listed senescence, mitochondrial dysfunction, transcriptional changes, sirtuins and reduced autophagy as candidate mechanisms contributing to podocyte ageing (Shankland et al. 2021). It is also considered that podocyte ageing is accelerated by age-related comorbidities such as obesity, diabetes and hypertension.

The importance of mTOR signalling in podocyte health is well documented (Canaud et al. 2013; Fantus et al. 2016; Gödel et al. 2011; Puelles et al. 2019a). McNicholas et al. demonstrated that mTOR signalling was a crucial maintenance factor in ageing animals, with global inhibition of mTOR signalling leading to greater levels of glomeruli with crescentic appearance (McNicholas et al. 2016). Anecdotal evidence of age-related changes in podocyte mTOR expression have been reported (Inoki et al. 2012). Data also suggest a role for mTOR signalling in both podocyte adaptation and the development and progression of glomerular disease (Nishizono et al. 2017; Zschiedrich et al. 2017). The present findings show that the number of mTOR + podocytes per glomerular cross-section increased significantly in control mice between 6 and 12 months, and again between 12 and 18 months. This points to a crucial role for mTOR in the successful adaptation of podocytes to physiological glomerular growth and/or normal ageing. Interestingly, following podocyte depletion at both 6 and 12 months, the number of mTOR + positive podocytes increased significantly, and this was associated with an adaptive increase in podocyte volume. Conversely, at 18 months of age, remaining podocytes were unable to undergo hypertrophic compensation in response to a mild podocyte depletion resulting in marked glomerular pathology. Interestingly, this decreased podocyte adaptive capacity at 18 months and subsequent glomerular pathology was associated with an inability to further elevate mTOR expression at this timepoint. This data is therefore supportive of the critical role of mTOR in adaptation of podocytes (Nishizono et al. 2017). Moreover, our findings highlight the relationship between decreased podocyte density and increased podocyte volume. This was evident in both control and DT-injected mice, with mTOR expression increasing at times of podocyte growth. Together these findings highlight the critical role for mTOR expression in both physiological and adaptive growth of podocytes in response to both ageing and podocyte injury. These data highlight that age may be a critical consideration when assessing a patient’s suitability for mTOR inhibition for the treatment of kidney diseases. Whilst this study highlights age-related differences in mTOR expression and podocyte adaptability, how this plays out in the setting of glomerular disease remains unknown and warrants further investigation.

As stated, our findings support previous reports that mTOR plays an important role in regulating podocyte hypertrophy in normal growth, healthy ageing and following podocyte loss. Physiological podocyte hypertrophy almost certainly involves increases in the number and/or length of major cytoplasmic processes, foot processes, filtration slits and slit diaphragms per podocyte. This adaptive response of podocytes is perhaps best highlighted in the present study by the quick onset and subsequent decrease in albuminuria following DT administration. At all ages, albuminuria peaked approximately 1–2 weeks following DT administration and then quickly decreased. In the youngest mice (1 month), albuminuria was no longer present 15 days post-DT. In mice aged 6, 12 and 18 months, albuminuria 56 days post-DT had decreased from the peak level by 87%, 85% and 77%, respectively. Our results demonstrated that hypertrophy of remaining podocytes occurred during this adaptation, but presumably generation and growth of new major processes, foot processes, filtration slits and slit diaphragms also occurred. Haemodynamic factors influencing the movement of water, ions and proteins are also re-setting during this adaptation period. Future studies analysing the growth of these podocyte structures and the molecular regulation of this growth in the healthy and ageing kidney are warranted.

Recent data from our laboratory indicate that podocyte hypertrophy may have a functional limit past which parietal epithelial cell (PEC) activation and glomerular pathology are induced (Puelles et al. 2019a). These findings supported previous reports from Wiggins et al. and Zschiedrich et al. who proposed a stage-based functional limit to podocyte hypertrophy and a narrow therapeutic window for effective mTOR inhibition in FSGS respectively (Wiggins et al. 2005; Zschiedrich et al. 2017).

We acknowledge that the present study has a number of limitations. Firstly, the oldest mice in this study were aged 18 months which is the equivalent of approximately 56 human years, or middle-age (Dutta et al. 2016). Thus, the present study did not address podocyte biology and adaptation in aged mice or mice equivalent to humans with advanced ageing. Another limitation was the use of synaptopodin immunofluorescence for estimating podocyte volume. Synaptopodin is mostly located in podocyte foot processes and thus may underestimate total podocyte volume.

## Conclusion

Glomerular and podometric indices change markedly with healthy ageing. However, our findings suggest that podocytes in control mice aged 18 months have compromised function and reduced capacity to adapt to mild podocyte depletion. Moreover, while remaining podocytes can increase their mTOR activity following podocyte depletion at younger ages, by 18 months of age they cannot and marked proteinuria and glomerulosclerosis ensue. Age-related changes in podocyte mTOR expression may influence the efficacy of mTOR inhibition in the treatment of podocytopathies.

## Supplementary information

Below is the link to the electronic supplementary material.

Supplementary file1 (TIF 1011 KB)

Supplementary file2 (TIF 1756 KB)

Supplementary file3 (TIF 334 KB)

Supplementary file4 (DOCX 20 KB)
