# Supplementary figures and images for: The ability of remaining glomerular podocytes to adapt to the loss of their neighbours decreases with age
Source: Cell Tissue Res. 2022 Mar 15;388(2):439–51. doi: 10.1007/s00441-022-03611-2 (PMC9035415; doi:10.1007/s00441-022-03611-2)

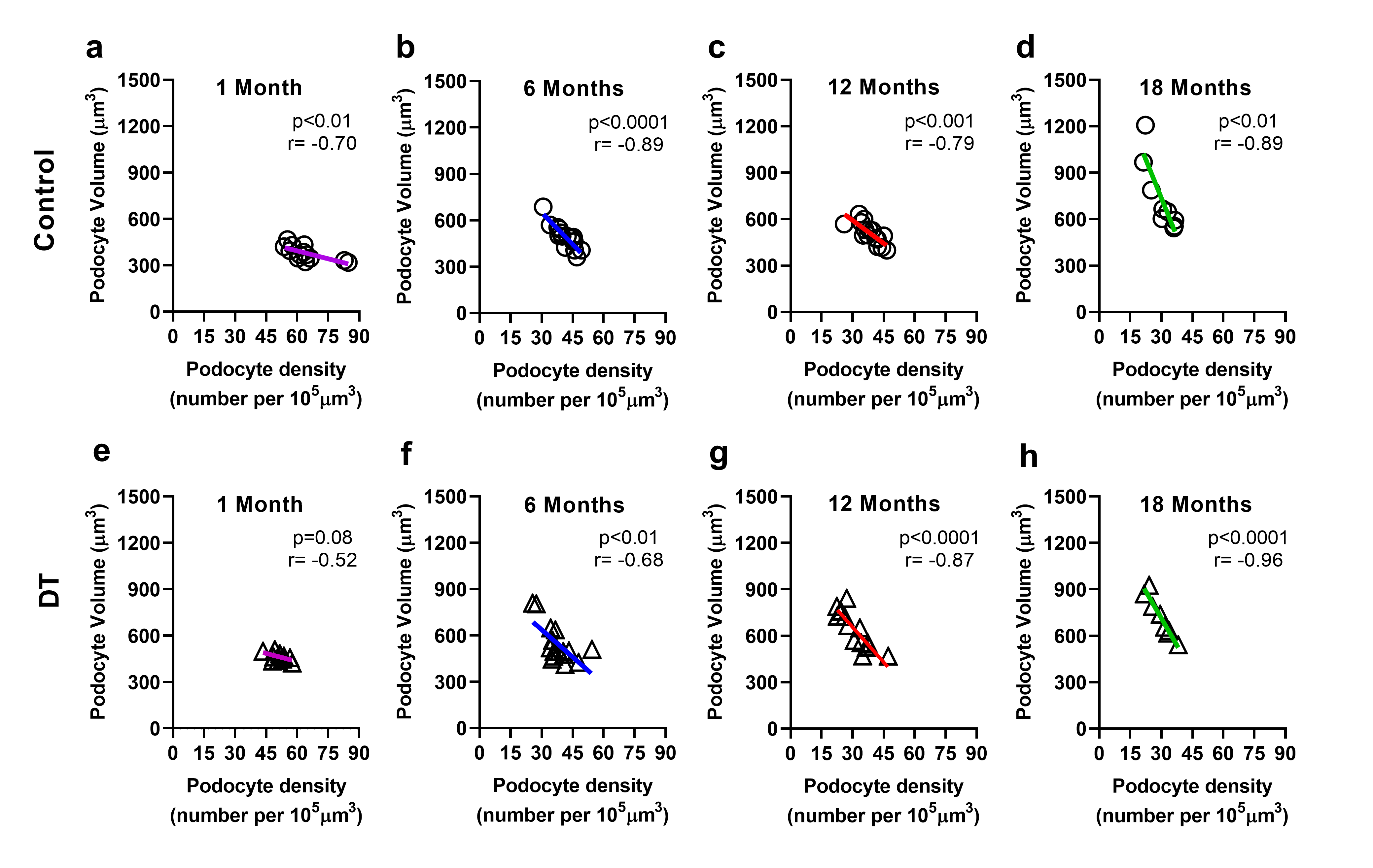

Supplement: Supplementary file 1 — Supplementary file1 (TIF 1011 KB) [file 441_2022_3611_MOESM1_ESM.tif]

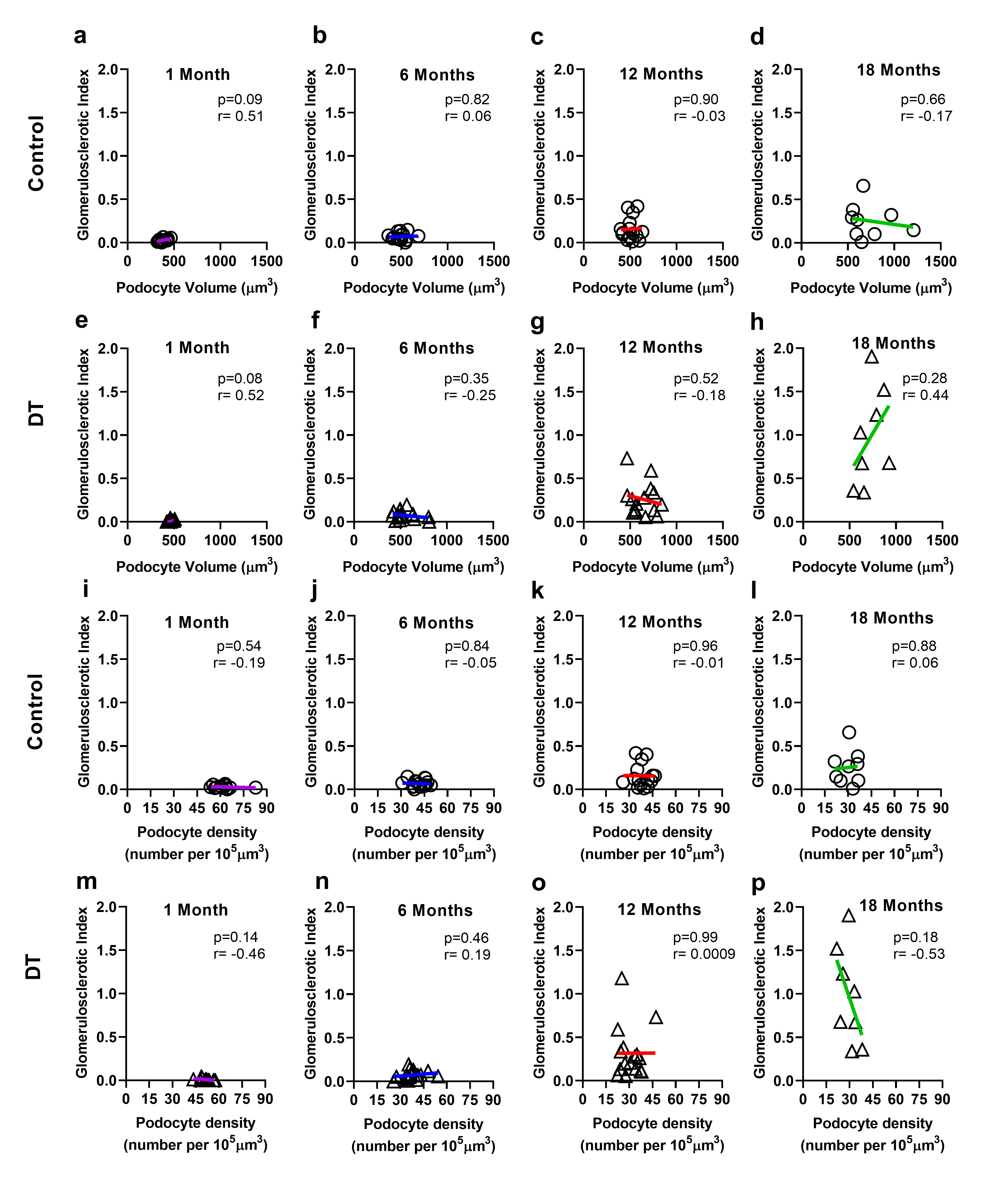

Supplement: Supplementary file 2 — Supplementary file2 (TIF 1756 KB) [file 441_2022_3611_MOESM2_ESM.tif]

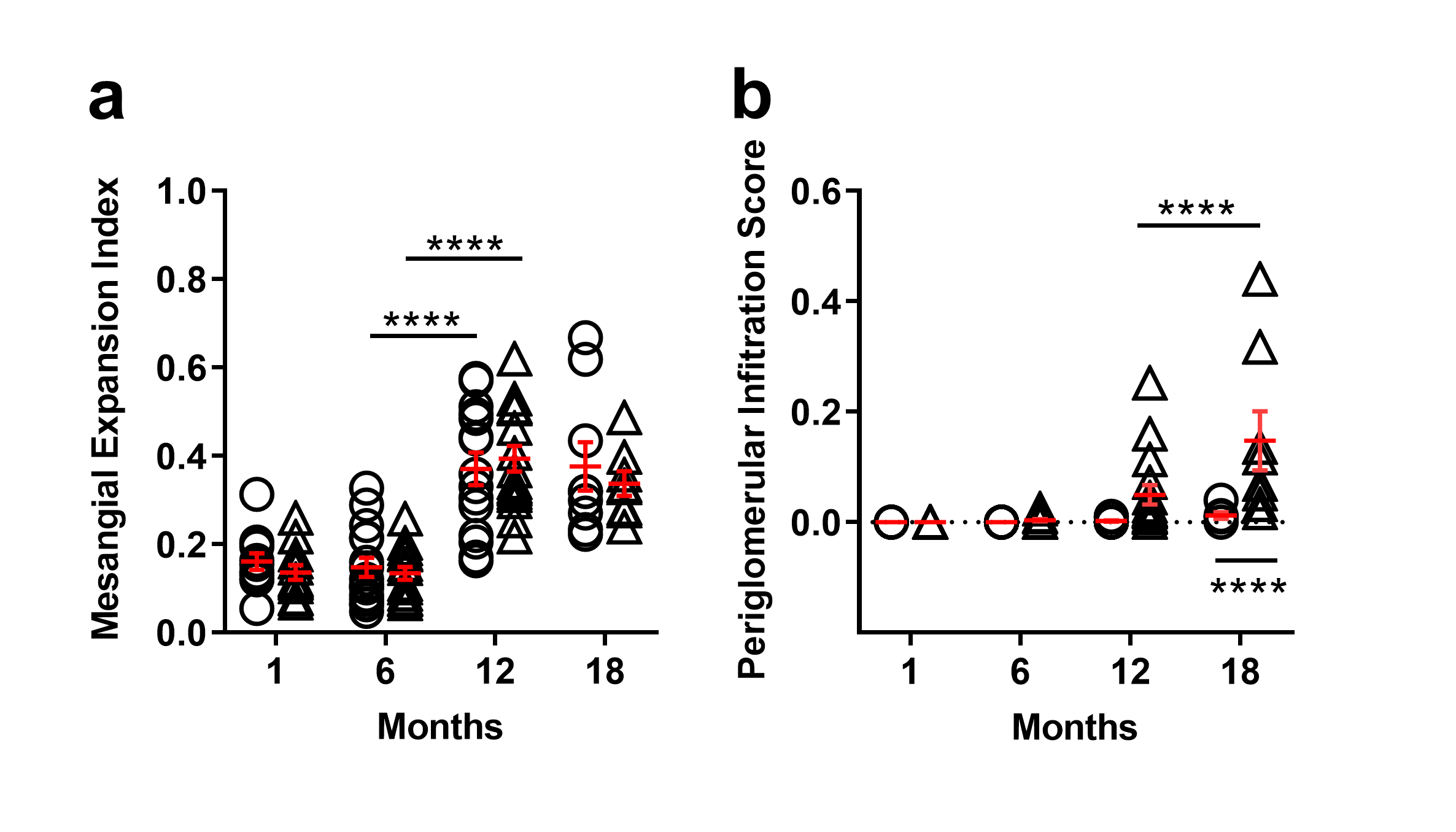

Supplement: Supplementary file 3 — Supplementary file3 (TIF 334 KB) [file 441_2022_3611_MOESM3_ESM.tif]
