# Supplementary material for: The ability of remaining glomerular podocytes to adapt to the loss of their neighbours decreases with age
Source: Cell Tissue Res. 2022 Mar 15;388(2):439–51. doi: 10.1007/s00441-022-03611-2 (PMC9035415; doi:10.1007/s00441-022-03611-2)
Supplement: Supplementary file 4 — Supplementary file4 (DOCX 20 KB) [file 441_2022_3611_MOESM4_ESM.docx]

**Supplementary Table 1: Body weight, kidney to body weight ratios and terminal blood glucose concentrations in control and DT-injected mice.** Mice were allocated to control or DT-treated groups by matching for bodyweight and fasting glucose at time 0. Data analysed by two-way ANOVA.

|  | 1 Month | | 6 Months | | 12 Months | | 18 Months | | p_age_ | p_treatment_ | p_age*treatment_ |
| --- | --- | --- | --- | --- | --- | --- | --- | --- | --- | --- | --- |
|  | **Control** | **DT** | **Control** | **DT** | **Control** | **DT** | **Control** | **DT** |  |  |  |
| Body Weight (g) | 26.68 ± 0.82 | 25.06 ± 0.92 | 41.95 ± 0.60 | 41.10 ± 0.66 | 46.88 ± 0.65 | 46.46 ± 0.62 | 48.43 ± 1.96 | 48.21 ± 1.26 | <0.0001 | 0.252 | 0.899 |
| Kidney to Body Weight Ratio (mg/g) | 9.98 ± 0.45 | 9.56 ± 0.27 | 7.03 ± 0.24 | 6.34 ± 0.18 | 7.06 ± 0.35 | 7.26 ± 0.37 | 7.12 ± 0.51 | 7.22 ± 0.37 | <0.0001 | 0.406 | 0.469 |
| Blood Glucose (mmol/l) | No Data | No Data | 9.86 ± 0.40 | 10.16 ± 0.41 | 9.64 ± 0.45 | 9.27 ± 0.37 | 9.99 ± 0.55 | 9.22 ± 0.70 | 0.635 | 0.395 | 0.658 |

**Supplementary Table 2:** Antibodies used for immunofluorescent staining.

| **Antibody** | **Dilution** | **Catalogue #** | **Company** |
| --- | --- | --- | --- |
| Polyclonal rabbit anti-mouse p57 antibody | 1 in 200 | SC8298 | Santa Cruz Biotechnology, Santa Cruz, CA |
| Polyclonal goat anti-mouse SNP | 1 in 400 | SC21537 | Santa Cruz Biotechnology, Santa Cruz, CA |
| mAb rabbit anti-phospho-s6- Ribosomal Protein | 1 in 50 | 5364S | Cell Signal Technology, Danvers, MA |
| Polyclonal rabbit anti-claudin 1 | 1 in 200 | 51-9000 | abcam, Cambridge, UK |
| mAb rat anti-CD44 | 1 in 100 | 5640S | Cell Signal Technology, Danvers, MA |
| Polyclonal donkey anti-rabbit Alexa Flour 555 | 1 in 200 | A31572 | Life Technologies, Carlsbad, CA |
| Polyclonal chicken anti-goat Alexa Flour 488 | 1 in 200 | A2467 | Life Technologies, Carlsbad, CA |

**Supplementary Table 3: Glomerular and podocyte morphometrics in control and DT-injected mice.** P values reflect two-way ANOVA, differences between groups depicted in Figure 2 following adjustment for multiple comparisons.

|  | 1 Month | | 6 Months | | 12 Months | | 18 Months | | p_age_ | p_treatment_ | p_age*treatment_ |
| --- | --- | --- | --- | --- | --- | --- | --- | --- | --- | --- | --- |
|  | **Control** | **DT** | **Control** | **DT** | **Control** | **DT** | **Control** | **DT** |  |  |  |
| Glomerular Volume (x10^5^ μm^3^) | 1.105 ± 0.05 | 1.144 ± 0.03 | 1.763 ± 0.06 | 1.652 ± 0.05 | 1.954 ± 0.07 | 2.085 ± 0.12 | 2.283 ± 0.10 | 1.986 ± 0.06 | <0.0001 | 0.303 | 0.071 |
| Podocyte Number | 69.08 ± 1.18 | 59.81 ± 0.77 | 73.69 ± 1.51 | 62.81 ± 1.27 | 77.33 ± 1.62 | 61.38 ± 1.71 | 73.00 ± 2.77 | 58.25 ± 2.71 | 0.012 | <0.0001 | 0.133 |
| Podocyte Volume (μm^3^) | 379 ± 12.99 | 462 ± 7.44 | 491 ± 19.26 | 507 ± 18.60 | 509 ± 17.50 | 662 ± 37.60 | 729 ± 74.4 | 721 ± 47.15 | <0.0001 | 0.007 | 0.038 |
| Podocyte Density (podocytes/10^4^ μm^3^ | 60.61 ± 1.27 | 51.5 ± 1.11 | 41.6 ± 1.25 | 37.9 ± 1.75 | 38.4 ± 1.39 | 30.8 ± 1.74 | 30.2 ± 1.99 | 29.7 ± 1.96 | <0.0001 | <0.0001 | 0.069 |

**Supplementary Table 4: Albumin to creatinine ratios in control and DT-injected mice.** Baseline are values at Day 0.

|  | 1 Month | | 6 Months | | 12 Months | | 18 Months | | p_age_ | p_treatment_ | p_age*treatment_ |
| --- | --- | --- | --- | --- | --- | --- | --- | --- | --- | --- | --- |
|  | **Control** | **DT** | **Control** | **DT** | **Control** | **DT** | **Control** | **DT** |  |  |  |
| Baseline (µg/mg) | 21.4 ± 5.5 | 21.5 ± 2.8 | 13.2 ± 1.9 | 15.2 ± 3.1 | 58.7 ± 15.5 | 47.7 ± 24.9 | 137.0 ± 54.4 | 196.0 ± 138 | 0.0008 | 0.630 | 0.815 |
| Peak (µg/mg) | 28.1 ± 5.4 | 81.2 ± 14.1 | 41.6 ± 8.64 | 921.4 ± 142.6 | 64.5 ± 10.8 | 799.3 ± 247.4 | 204.1 ± 59.7 | 3219 ± 704.5 | <0.0001 | <0.0001 | 0.0004 |
| Terminal (µg/mg) | 19.2 ±2.9 | 20.2 ± 4.5 | 33.9 ± 6.5 | 110.7 ± 13.73 | 42.6 ± 6.5 | 185.4 ± 29.76 | 64.4 ± 21.7 | 640.1 ± 222.4 | <0.0001 | <0.0001 | 0.0002 |
| Area Under the Curve | 279 ±49 | 781 ± 177 | 1211 ±298 | 12202 ± 2704 | 1155 ± 308 | 17470 ± 7214 | 6384 ± 2155 | 87892 ± 19718 | <0.0001 | <0.0001 | <0.0001 |
